# Divide and Conquer: Functional Segregation of Synaptic Inputs by Astrocytic Microdomains Could Alleviate Paroxysmal Activity Following Brain Trauma

**DOI:** 10.1371/journal.pcbi.1002856

**Published:** 2013-01-24

**Authors:** Vladislav Volman, Maxim Bazhenov, Terrence J. Sejnowski

**Affiliations:** 1 Howard Hughes Medical Institute, Computational Neurobiology Laboratory, The Salk Institute for Biological Studies, La Jolla, California, United States of America; 2 Center for Theoretical Biological Physics, University of California at San Diego, La Jolla, California, United States of America; 3 L-3 Applied Technologies/Simulation, Engineering, & Testing, San Diego, California, United States of America; 4 Department of Cell Biology and Neuroscience, University of California at Riverside, La Jolla, California, United States of America; 5 Division of Biological Sciences, University of California at San Diego, La Jolla, California, United States of America; Massachusetts Institute of Technology, United States of America

## Abstract

Traumatic brain injury often leads to epileptic seizures. Among other factors, homeostatic synaptic plasticity (HSP) mediates posttraumatic epileptogenesis through unbalanced synaptic scaling, partially compensating for the trauma-incurred loss of neural excitability. HSP is mediated in part by tumor necrosis factor alpha (TNFα), which is released locally from reactive astrocytes early after trauma in response to chronic neuronal inactivity. During this early period, TNFα is likely to be constrained to its glial sources; however, the contribution of glia-mediated spatially localized HSP to post-traumatic epileptogenesis remains poorly understood. We used computational model to investigate the reorganization of collective neural activity early after trauma. Trauma and synaptic scaling transformed asynchronous spiking into paroxysmal discharges. The rate of paroxysms could be reduced by functional segregation of synaptic input into astrocytic microdomains. Thus, we propose that trauma-triggered reactive gliosis could exert both beneficial and deleterious effects on neural activity.

## Author Summary

Homeostatic plasticity refers to the ability of neurons and neuronal circuitry to adjust their properties in order to maintain physiologically relevant electrical activity notwithstanding perturbations in synaptic input. Synaptic input is often chronically reduced immediately following brain trauma, and previous studies had suggested that homeostatic synaptic plasticity can aid in the dynamical transition of the traumatized network toward epileptic seizures, a condition known as “post-traumatic epilepsy”. This form of homeostatic plasticity is mediated by glial cells which release regulatory molecules shortly after trauma. In this study we used computational modeling to investigate the mechanisms and the implications of glial mediated plasticity early after trauma. We show that astrocytes (a subtype of glial cells) exert both beneficial and deleterious effects on post-traumatic reorganization of neural activity. This suggests that, in the dysfunctional neuronal network, some aspects of glial-neuronal signaling could alleviate the dynamical transition to pathological activity.

## Introduction

Post-traumatic epilepsy develops in some but not all head injury cases, depending on the severity of injury and the time elapsed since trauma. Often there is a latent period between the traumatic event and onset of paroxysmal activity [1]. Identification of neurological mechanisms underlying this latency to seizures can offer a possibility for therapeutic intervention. Experimental and modeling studies suggest that this slow transition from normal to paroxysmal activity might depend on homeostatic adjustment of synaptic conductances, connectivity and intrinsic excitability properties [2], [3].

Homeostatic synaptic plasticity (HSP) likely operates on several spatial and temporal scales [4]. Chronic synaptic and neuronal inactivity, such as the one that often occurs following trauma, engages glial cells to release tumor necrosis factor alpha (TNFα) [5], [6], [7]. This relatively slow process (global effects in culture are measurable after ∼48 hours of inactivity [6]) may represent a global “network response” to prolonged inactivity [8]. The strengthening of inputs from the open eye during monocular deprivation is another slow process that is mediated by TNFα [7], [8], [9]. Early after trauma elevated levels of TNFα are likely to be spatially localized to their glial sources, implying spatial localization of homeostatic synaptic plasticity.

Earlier studies showed that TNFα causes a rapid, p55 receptor mediated insertion of neuronal AMPA receptors [10], and endocytosis of GABA receptors [6]. Thus, TNFα could promote epileptogenesis by shifting the excitation-inhibition balance in favor of excitation. Consistent with this, systemic administration of TNFα [11] and constitutive over-expression of TNFα [12] had pro-epileptic effects. Seizure incidence was dramatically reduced in knockout mice lacking p55 TNFα receptors [13], [14]; susceptibility to seizures was reduced following systemic pre-injection of TNFα antibodies [15]. These data suggest that TNFα can promote epileptogenesis [16]. Given the role of TNFα in HSP [7], [8], [9], the implication is that homeostatic synaptic plasticity can drive the traumatized network toward epileptic activity [3], [17], [18], [19].

In our previous studies [3], [18] we showed that trauma-triggered HSP can transform cortical activity from asynchronous spiking (∼5 Hz for pyramidal neurons, ∼10 Hz for inhibitory neurons) to paroxysmal bursting, and we further showed that the pattern of trauma changes the threshold for epileptogenesis [20]. In those studies we implicitly assumed that HSP represents the action of TNFα which is released in response to chronically low levels of neuronal activity incurred by the traumatic injury. We also assumed that HSP adjusted synaptic conductances in a manner that depended on the network-global averaging of neuronal activity. The assumption of global network averaging of neuronal activity is likely to be valid at sufficiently long time after trauma, when levels of TNFα had equilibrated throughout the network. However, at a short time (several hours) after trauma, elevated levels of TNFα are likely to be localized around their glial sources [21], thus implying spatial localization of HSP and spatially heterogeneous disruption of excitation-inhibition balance that may strongly favor the transition to seizures. Given the extensive evidence for the dramatic involvement of TNFα in post-traumatic epilepsy [16], high levels of localized TNFα a short time after brain injury [21] are not consistent with the relatively low incidence of paroxysmal spikes and seizures during that period.

In the present study, we addressed this question by studying the early effects of TNFα mediated HSP hours after trauma. Homeostatic synaptic plasticity restored the average network firing rate to its pre-traumatic level but transformed asynchronous spiking to paroxysmal bursts. Thus, we adopted the rate of paroxysmal burst generation (rather than the network-averaged firing rate) as a measure of network's propensity to exhibit the transition to seizures. Paroxysmal bursts of highly correlated population activity in our model resembled interictal epileptiform discharges (IEDs), which are often considered an important diagnostic feature of epileptic seizures [22], [23], [24]. Thus, a higher rate of population bursting in a post-traumatic model network was considered an indicator of stronger propensity to seizures. With spatially constrained HSP (“local HSP”), representing local synaptic scaling by TNFα, paroxysmal bursts occurred in post-traumatic network at a high rate, with little dependence on the fraction of deafferented neurons (trauma volume). This was in striking contrast to the gradual dependence of burst rate on trauma volume that characterized the later stage of “global HSP”. Properties of paroxysmal discharges could be modulated by functional segregation of synaptic inputs into reactive astrocytic microdomains [25], [26]. Thus, our modeling studies suggest that some aspects of reactive astrogliosis might alleviate paroxysmal activity early after trauma.

## Results

The primary goal of the present study was to explore the impact of spatially localized homeostatic synaptic plasticity (HSP) on the emergence of paroxysmal activity in deafferented post-traumatic cortical network and to identify mechanisms that might operate to suppress this activity in the early post-traumatic window. We have shown previously that the spatial pattern of trauma itself can significantly affect the trauma threshold for paroxysmal burst generation [20]. In addition, as we show below, segregated synaptic regulation by astrocytic microdomains can also affect post-traumatic electrical activity.

### Comparative design of the HSP models

Homeostatic regulation is likely to operate on a localized spatial scale during the early phase of response to trauma, reflecting the local response of glial cells to nearby synaptic activity. Such a change of scale could in principle affect our earlier conclusions regarding the role of trauma pattern in post-traumatic epileptogenesis [20]. In particular, in our earlier studies [20] we found that the trauma threshold for the emergence of paroxysmal events in post-traumatic network depended on the pattern of trauma itself. In those studies, the extent of trauma was parameterized by the fraction of deafferented model neurons,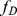, which we will refer to as the “volume of trauma”. When burst rate was plotted vs. the trauma volume parameter, focal trauma (spatially contiguous set of deafferented neurons) caused lower burst threshold as compared to diffuse trauma (spatially randomly distributed set of deafferented neurons). Thus, it was important to validate the conclusions of our earlier studies regarding the role of trauma spatial organization in generation of post-traumatic paroxysmal activity.

Here, we assumed that the downregulation of excitatory synaptic conductances in a computational model of a hyperactive pyramidal (PY) neuron was determined by the time-averaged firing rate of the postsynaptic neuron, consistent with postsynaptic synaptic scaling [4]. On the other hand, upregulation of excitatory synaptic input in response to reduced levels of synaptic activity was determined in our model by the time-averaged firing rate of all PY neurons that projected their synapses to a PY neuron under consideration, corresponding to glial scaling of synaptic conductances by TNFα [8]. Thus, the baseline model is described by “local UP” regulation and “local DOWN” regulation of synaptic conductance. This model is referred to below as a *local HSP model*.

To compare with our previous results [20] we also used a global HSP model, in which both pre- and postsynaptic components of homeostatic synaptic scaling were determined by the global, network-averaged, firing rate of model PY neurons. Thus, the “global HSP” model that was used in our previous studies [20] is described by “global UP” regulation and “global DOWN” regulation of synaptic conductance. A shift in the spatial scale of HSP rule in the present model would correspond to the transition from the early phase of post-traumatic reorganization (during which upregulation of synaptic conductance is constrained to glial sources of TNFα) to the later phase (of equilibrated levels of TNFα).

**Figure 1:**
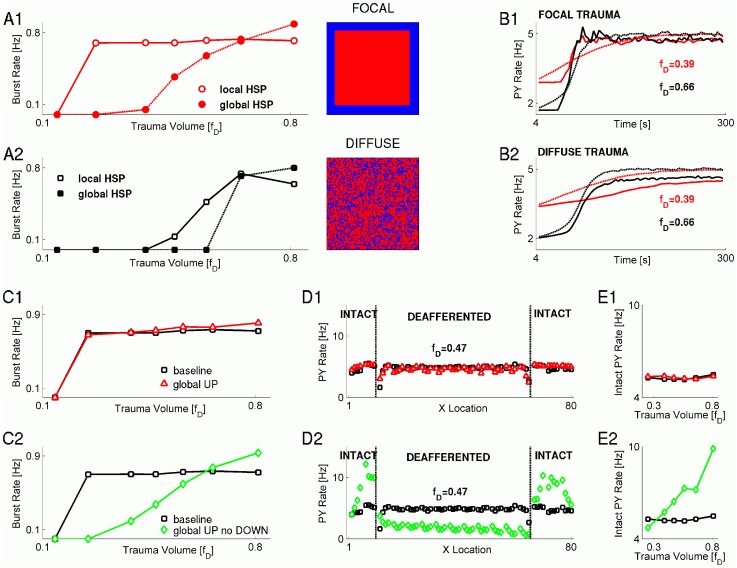
Spatial pattern of deafferentation affects post-traumatic reorganization of activity. **A** Burst rate vs the trauma volume, for focal (**A1**) and diffuse (**A2**) trauma scenarios and different schemes of HSP: local HSP (open red circles and open black squares); “global HSP” (closed red circles and closed black squares). **B** Network-averaged firing rate of PY model neurons vs the time since deafferentation, for focal (**B1**) and diffuse (**B2**) trauma pattern scenarios. Red: small trauma volume (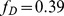). Black: larger trauma volume (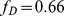). Solid lines: Local HSP scheme. Dashed lines: Global HSP scheme. **C1**: Burst rate vs trauma volume for baseline model (squares) and the model in which the upregulating component of HSP averaged over global network activity (triangles). **C2**: Burst rate vs trauma volume for baseline model (squares) and the model in which downregulating component of HSP was omitted and upregulating component of HSP averaged over global network activity (open diamonds). All scenarios considered are for focal trauma. **D1**: Averaged PY firing rate vs neuronal location in the network (left plot), and network averaged PY firing rate vs trauma volume (right plot). Keys are the same as in **C1**. **D2**: Averaged PY firing rate vs neuronal location in the network (left plot), and network averaged PY firing rate vs trauma volume (right plot). Keys are the same as in **C2**. Boundaries between traumatized and intact regions are demarcated with dashed lines. **E1** Averaged firing rate of intact PY neurons at the boundary between deafferented and intact regions, vs the trauma volume, for scenarios shown in **C1**. Keys are the same as in **C1**. **E2**: Averaged firing rate of intact PY neurons at the boundary between deafferented and intact regions, vs the trauma volume, for scenarios shown in **C2**. Keys are the same as in **C2**. In **D–E**, trauma volume parameter was 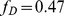.

In some simulations (e.g., Figure 1C) we only changed the spatial scale of presynaptic, upregulating, HSP component from local (averaged only over those PY neurons that project their synapses to a given neuron, as in “local HSP” or baseline model) to global (averaged over all PY neurons in the network, “global UP” model in Figure 1C). Note that the “global UP” model differs from “global HSP” model in that in the former the downregulating postsynaptic HSP component is local (i.e., based on the activity of the specific postsynaptic neuron, similar to the baseline model). In other simulations, the downregulating postsynaptic HSP component was removed altogether from the model network, to assess the impact that homeostatic downregulation of synaptic conductance might have on collective activity; this model is referred to as “global UP no DOWN”. Note again that the “global UP no DOWN” model differs from the “global HSP” model in that in the latter the downregulating component of HSP is present. In yet other simulations, the set of synaptic inputs in the local HSP scheme was further randomly and evenly partitioned into several groups, for each of which we applied equations that described presynaptic component of HSP (Materials and Methods). Such partitioning into sub-groups of synaptic inputs was taken to mimic partition of the cortex into astrocytic microdomains [25], [27]. Finally, we compared two different patterns of trauma: *focal* trauma (in which a spatially contiguous subpopulation of neurons was deafferented, (e.g., Figure 1A1)), and *diffuse* trauma (in which deafferentation affected fraction of neurons randomly selected from the entire network (e.g., Figure 1A2)). In what follows, the “baseline” model network configuration is defined as a network with one microdomain per neuron, local HSP rule, and subject to focal trauma.

### The threshold for post-traumatic epileptogenesis depends on the pattern of trauma

Within the “early response” scheme of the HSP based on the activity of presynaptic neurons (local HSP, see above and Materials and Methods), the rate of paroxysmal bursts was significantly higher for focal trauma (Figure 1A1) compared to the spatially diffuse trauma (Figure 1A2), and this distinction was observed over a wide range of trauma volume parameter values, 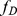. This was generally consistent with earlier studies in which we showed that the spatial pattern of trauma could critically affect the threshold for post-traumatic paroxysmal activity [20]. In Figures 1A1, 1A2, we also plotted the results obtained with the global HSP model (in which both up and down regulation of synaptic conductance was determined based on the global network-wide average over activities of pyramidal neurons), to compare them with the present model, which made use of local HSP. Although the two models produced the same result qualitatively (both showed an increase in the rate of paroxysmal activity above some critical threshold of trauma volume parameter, and in both cases the threshold was higher for the diffuse trauma), the threshold for paroxysmal activity appeared to be much lower in the case of local HSP rule.

The difference between the two HSP models was also reflected in the dynamics of the network-averaged firing rate of the PY neurons, shown in Figure 1B1 for the case of focal trauma. In the local HSP model of the focal trauma, and for relatively small trauma volumes (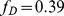), the network-averaged firing rate of the pyramidal neurons showed a much steeper transition to its post-traumatic target value compared to the more gradual change observed within the global HSP model (Figure 1B1, compare solid red lines for local HSP model and dashed red lines for global HSP model). In contrast, after more severe trauma (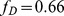) the approach of the network-averaged firing rate of the model PY neurons toward its post-traumatic target value was more similar for both the local and global HSP scenarios (Figure 1B1, black solid and black dashed lines for local and global HSP, respectively).

The quantitative differences between the two HSP models, as reflected in the dynamics of the network-averaged firing rate of model pyramidal neurons, were discernible also within the scenario of diffuse trauma (Figure 1B2). However, in the diffuse trauma scenario, there was no qualitative difference between the firing rate reorganization dynamics in different HSP models; both local and global HSP models caused gradual recovery of firing rate for relatively small trauma volume (Figure 1B2, solid red and dashed red for local and global HSP models, 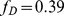) and steeper transition in the case of more severe trauma (Figure 1B2, solid black and dashed black for local and global HSP models, 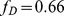).

Thus, qualitatively, the strongest effect of HSP localization on the post-traumatic reorganization of electrical activity was observed for relatively small volumes of focal trauma. This is consistent with the results in Figures 1A1, 1A2, which show that the primary effect of local HSP is to lower the trauma volume threshold for burst generation and that this effect is more pronounced in the focal trauma scenario.

### Independence of the burst rate on the trauma volume in a local HSP model

We next explored the intriguing independence of the paroxysmal burst rate on trauma volume in a model with a local HSP rule. Several mechanisms in the new model of homeostatic plasticity could have been responsible for this observation. It could have been a consequence of normalizing the neuronal firing rates, which was implemented by downregulating postsynaptic component of HSP (based on the firing rate of postsynaptic neuron, as suggested in [4]). Alternatively, the independence of burst rate on the trauma volume could follow from the local scale of the presynaptic component of the HSP, in contrast to the global, network-wide, scale of HSP employed in earlier models of late post-traumatic phase [3], [18], [20]. To test this second possibility, we replaced the local scale of the presynaptic HSP (for which the averaging of firing rates was performed over the set of those model PY neurons that projected synapses to a given neuron) with the global scale of the presynaptic HSP (for which the averaging of firing rates was performed over all model PY neurons). The postsynaptic component of HSP remained local and was determined by the firing rate of the postsynaptic neuron. As shown in Figure 1C1, this manipulation on the spatial scale of the presynaptic HSP component did not result in any significant effect on the burst rate – trauma volume relation.

We then tested the possibility that the apparent independence of burst rate on trauma volume was dominated by the postsynaptic downregulating component of HSP. When the postsynaptic component of HSP was excluded from the model and the global scale scheme was used for the presynaptic component, the linear relation (in the supra-threshold regime) between the burst rate and trauma volume was recovered (Figure 1C2, open green diamonds). Thus, it appeared that the downregulating postsynaptic component of HSP acted as a permissive factor, either allowing or preventing the burst rate to be modulated by the spatial scale of the presynaptic HSP component.

### Spatial distribution of PY firing rates after HSP

Earlier studies [3] suggested that post-traumatic paroxysmal activity arises because HSP acts to restore the firing rates of pyramidal neurons to their pre-traumatic value. Thus, we reasoned that the above dependence of burst rate on trauma volume during early stages of post-traumatic reorganization might, at least partially, be reflected in the firing rates of PY neurons. Thus, we estimated the dependence of pyramidal firing rate on the neuronal location in the network. For this analysis, neuronal firing rates were sampled from the center-symmetric strip (cross section was 5 cells from the center of the strip) of “neural tissue”, and at each point the firing rates of model PY neurons were averaged over the cross-section of the sampled strip.

With the postsynaptic downregulating component of HSP present, the firing rate of PY neurons (either deafferented or intact) was clamped at ∼5 Hz and did not depend on the spatial scale of presynaptic HSP component (Figures 1D1 and 1E1). Indeed, the postsynaptic HSP component prevented firing rate of any individual PY neuron to exceed its preset target rate. The firing rate of the intact neurons never increased and the firing rate of the deafferented neurons reached the target regardless of the presynaptic HSP model. Therefore the model with global presynaptic scaling was virtually indistinguishable from the baseline model (with focal trauma and local HSP) and for both models the firing rate was independent on the trauma volume (Figures 1E1).

When the global scale presynaptic scheme was combined with exclusion of the postsynaptic downregulating HSP component, the averaged firing rate of intact PY neurons at the traumatized-intact boundary — defined as a set of PY neurons located within one synaptic footprint from the boundary deafferented neuron (see Materials and Methods) — displayed strong dependence on the trauma volume parameter 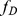 (Figure 1E2, open green diamonds). In this model, increase of the trauma volume led to an increase in the size of the deafferented (less active) PY population and, therefore, required a stronger increase in the firing rate of the intact PY population to keep the overall firing rate constant. This model allowed an increase because the postsynaptic downregulating HSP component was absent. Therefore, for intermediate and high trauma volumes 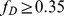 (Figure 1E2), the firing rates of intact PY neurons were higher than those in the baseline model (with focal trauma and local HSP) and the firing rates of deafferented neurons were lower (Figures 1D2, black squares vs. green diamonds). Surprisingly, for intermediate trauma volumes 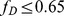 (Figure 1C2, black squares vs. green diamonds) the relation between the corresponding rates of paroxysmal bursts was opposite to the one observed for neuronal firing rates (i.e., for intermediate values of the trauma volume parameter the burst rate in the “global UP no DOWN” model was lower than the burst rate in the baseline model with focal trauma and local HSP). This suggests that the burst rate was limited by the firing rates of deafferented neurons; even when intact neurons fired at higher rate, they only occasionally triggered bursts in the deafferented population. Thus, although the “firing rate–burst rate” relation hypothesis could qualitatively account for the dependence of burst rate on trauma volume, it failed to explain the quantitative differences between the two HSP scenarios (local vs. global models).

### Paroxysmal discharges are generated by the intact pyramidal neurons located at the intact-deafferented boundary

**Figure 2:**
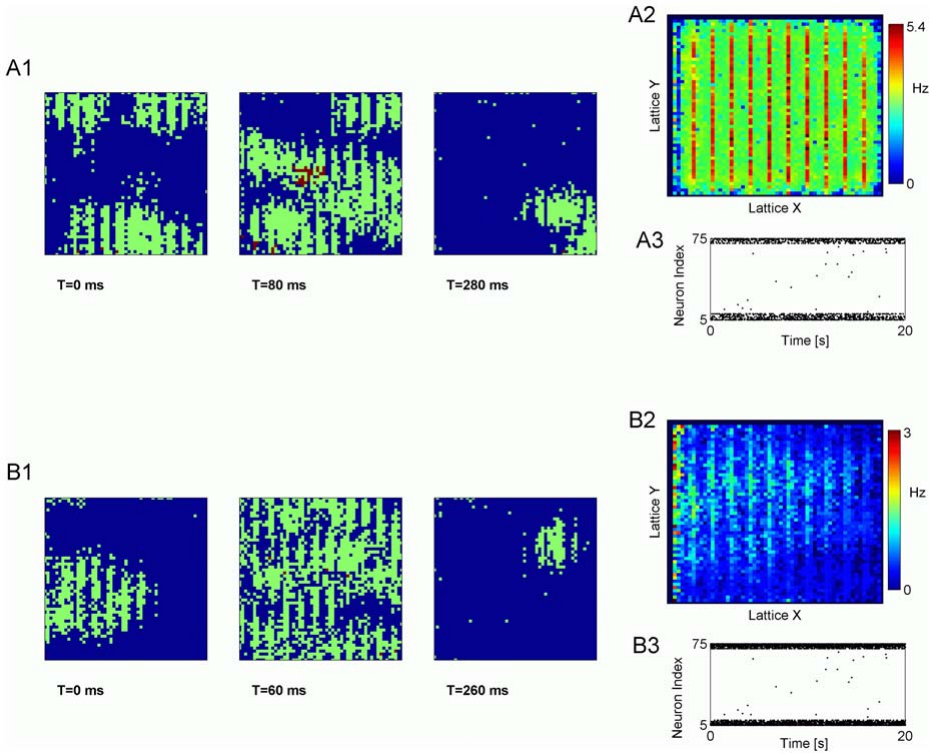
Spatio-temporal patterns of collective activity in traumatized networks. **A** **A1** Snapshots of collective activity in deafferented region of model network with local HSP scheme. Each pixel represents spiking activity of a neuron averaged over a time window of T = 20 ms. Color code is cool for low firing rate and warm for higher firing rate. Image boundaries correspond to the boundary between the intact and deafferented zones. **A2** Time-averaged (T = 50 s) firing rate of model neurons in network with local HSP scheme. Axes are spatial dimensions of network grid. Firing rate averaging is done after the network had reached its post-traumatic steady state. **A3** Sample raster plot of activity in network (local HSP scheme) in which synaptic connectivity between deafferented and intact parts was destroyed. **B** **B1** Snapshots of collective activity in deafferented region of model network with global HSP scheme. Each pixel represents spiking activity of a neuron averaged over a time window of T = 20 ms. Color code is cool for low firing rate and warm for higher firing rate. Image boundaries correspond to the boundary between the intact and deafferented zones. **B2** Time-averaged (T = 50 s) firing rate of model neurons in network with global HSP scheme. Axes are spatial dimensions of network grid. Firing rate averaging is done after the network had reached its post-traumatic steady state. **B3** Sample raster plot of activity in network (global HSP scheme) in which synaptic connectivity between deafferented and intact parts was destroyed. In all panels, trauma volume parameter was 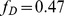.

In our previous studies we showed that, in the deafferentation model of cortical trauma, paroxysmal activity is generated by the intact pyramidal neurons located at the boundary between intact and deafferented regions [20]. Because our previous studies utilized “global HSP” model, it was important to test whether or not the same conclusions would hold as well for networks with “local HSP” scenario. Figures 2A1, 2B1 show snapshots of spatial activity in deafferented regions of model networks (trauma volume parameter 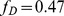), for “local HSP” (Figure 2A1) and “global HSP” (Figure 2B1), and in both scenarios it is seen that paroxysmal activity propagates in a wave-like manner, from intact and into the deafferented part of the network (in Figures 2A1, 2B1 image boundaries correspond to the boundary between the intact and deafferented regions of a network, so that only the dynamics in deafferented regions are shown). In Figures 2A2, 2B2 the spatial spread of activity is further quantified by computing and plotting, for two scenarios, the time-averaged firing rates of the model neurons. In these plots, the axis are the spatial dimensions of the network grid, and color codes the firing rate of individual neurons, averaged over a long time window (T = 50 s) after the network had reached its post-traumatic steady state. Destruction of synaptic connectivity between the intact and deafferented parts of the network completely eliminated paroxysmal activity (Figures 2A3, 2B3). This confirms that paroxysmal activity in the deafferented part of the network critically depends on the existence of functional synaptic connectivity with the intact part.

### Post-traumatic paroxysmal discharges are modulated by the spatial scale of synaptic connectivity and HSP scheme

In our previous studies [28] we showed that the rate of post-traumatic paroxysmal bursts may be set not only by the firing rates of intact PY neurons; other determinants of collective activity include the spatial distribution of intact PY neurons and the strengths of their recurrent synapses [28]. This implies that the spatial scale of synaptic connectivity pattern — the spatial extent to which synaptic connections can be formed, as determined by the size of the synaptic footprint (see Materials and Methods for details) — might predispose the traumatized network to become more or less epileptogenic. Indeed, experimental evidence suggests that, following traumatic brain injury, a network is likely to undergo changes in its anatomical connectivity [17], [19], [29], [30]. Further, modeling studies showed that these changes in anatomical connectivity could breach the excitation-inhibition balance and generate epileptic-like seizures [2]. Although reorganization of synaptic connectivity likely occurs on much slower time scales than the ones we study here (days vs. hours), rapid and localized remodeling of synaptic connections was also reported [31].

**Figure 3:**
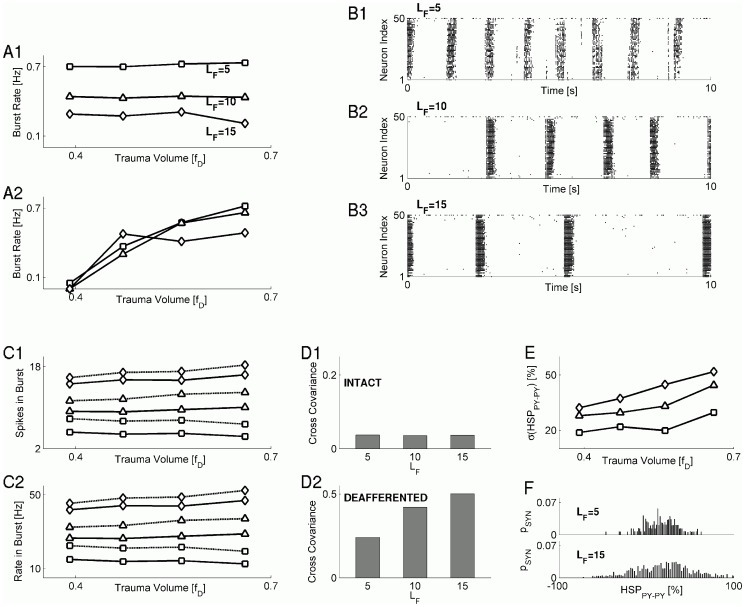
Characteristics of synaptic connectivity modulate the properties of paroxysmal discharges. **A** Burst rate vs the trauma volume for different sizes of synaptic footprint, for local HSP (**A1**) and “global HSP” (**A2**) models: the model with 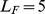 (squares); 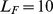 (triangles); 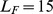 (diamonds). **B** Raster plots exemplifying network dynamics in post-traumatic state for different sizes of synaptic footprint: 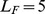 (**B1**); 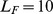 (**B2**); 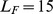 (**B3**). **C** Averaged number of spikes per neuron per burst (**C1**) and averaged firing rate per neuron per burst (**C2**), vs the trauma volume. Keys are the same as for panel **A**. Solid lines: PY neurons. Dashed lines: IN neurons. **D** Averaged cross-covariance between model PY neurons in intact subnetwork (**D1**), and model PY neurons in deafferented subnetwork (**D2**), for different sizes of synaptic footprint. **E** Standard deviation of the distribution of HSP scaling factors at PY-PY sy apses vs the trauma volume, for different sizes of synaptic footprint. Keys are the same as for panel **A**. **F** Examples of HSP scaling factor distributions (fraction of synapses 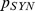 plotted vs. the actual value of synaptic scaling factor, 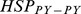) for different synaptic footprint sizes: 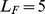 (top), 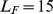 (bottom).

We addressed the possible interplay of synaptic connectivity and HSP spatial scales by scaling up the size of synaptic footprint in the model. The synaptic footprint is the region from and to which a given model neuron could receive or send synaptic connections, and in the model network it was a 10×10 square centered at the neuron under consideration. As the size of synaptic footprint was scaled (by scaling the dimensions of the square region from and to which synapses could be received/sent), the probability of establishing synaptic contacts inversely depended on the number of potential pre-synaptic partners, as determined by the footprint size, in order to keep the average number of synapses to a given neuron the same, regardless of the footprint size. This allowed us to avoid conflating the effects of footprint size with an increase in synaptic connectivity. As Figure 3A1 shows, the rate of paroxysmal burst generation was smaller for larger synaptic footprint sizes (parameterized as the half-length 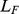 of square side). This reduction in burst rate was paralleled by an increase in the rate of neuronal firing during the burst, which in turn stemmed from an increase in the number of spikes fired during the burst (Figures 3B1–3 for sample burst profiles, Figures 3C1,2 for quantification of intra-burst spiking activity). By contrast, within the global HSP scheme, manipulations of the synaptic footprint size averaging activity over all model PY neurons had a much weaker effect on the burst-rate trauma-volume relation (Figure 3A2).

Because burst nucleation in our model required the activity of a certain fraction of intact neurons to be sufficiently correlated (in order to be able to “ignite” their deafferented postsynaptic partners) [28], a reduction in the rate of paroxysmal discharge could signal reduced correlation between burst-triggering intact neurons. However, correlation between activities of intact neurons on the boundary did not exhibit any remarkable dependence on the size of synaptic footprint (Figure 3D1). The correlation between activities of deafferented neurons did grow up with the increasing size of synaptic footprint (Figure 3D2). Thus, the reduction in burst rate that was observed for a larger synaptic footprint did not depend on the reduced correlation between burst-igniting neurons.

Another possibility is that a reduced rate of burst generation reflects higher heterogeneity in interconnectedness and synaptic weights for synapses formed among neurons on the boundary between intact and deafferented regions. Indeed, distributions of HSP scaling factors at PY-PY synapses in post-traumatic steady state (Figure 3F) were characterized by larger standard deviations for scenarios with larger footprint sizes (Figure 3E). These results suggest that heterogeneity of interconnectedness and synaptic conductances at the intact-deafferented boundary could help to alleviate the onset of paroxysmal bursting activity, but this comes at the expense of more intense spiking activity during the burst.

### Segregated regulation of synaptic input by astrocytic microdomains can decrease the rate of paroxysmal discharges

Assuming that the heterogeneity of synaptic organization at the boundary between traumatized and intact regions is likely to be important in post-traumatic epileptogenesis, we sought to identify the physiological mechanisms that might mediate this effect. Experimental evidence suggests that homeostatic scaling of synaptic conductances might be at least in part mediated by the soluble tumor necrosis factor alpha (TNFα) that is believed to be released from astrocytes to compensate for low levels of glutamatergic synaptic activity [8]. It is well established that astrocytes can sense glutamatergic synaptic activity and respond to it with diverse spatio-temporal patterns of free cytosolic calcium [32], [33]. Although a typical astrocyte contacts ∼100,000 synapses [34], in a recent study, the calcium-mediated detection and modulation of synaptic release by astrocytes under physiological conditions was local, with regulation occurring independently along astrocytic processes (branches) in groups of 10 s of adjacent synapses [27]. These findings are consistent with the notion of astrocytic microdomains, with each microdomain responsible for the autonomous regulation of a small cluster of spatially proximal synapses [25]. Note that microdomains are morphological feature of astrocytes, and thus astrocytic microdomain should not necessarily contact synapses for regulation; however, any synapse that is regulated by an astrocyte belongs, by definition, to unique astrocytic microdomain.

Spatial localization of astrocytic signaling may translate into autonomous regulation of groups of synapses. Since we consider here the early stage of post-traumatic response (when a relatively high glial TNFα concentration is likely to be spatially constrained to its release sites) such autonomous regulation could increase the heterogeneity of synaptic conductances scaled by glia-mediated HSP. Thus, we hypothesized that functional segregation of synaptic inputs into astrocytic microdomains could help alleviate the rate of paroxysmal discharges in our model networks. To test this hypothesis, for each model PY neuron the set of all 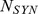 collateral synapses to it (from PY and IN neurons) was randomly partitioned into 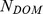 groups (microdomains), such that on average a group of 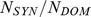 synapses constituted a microdomain). Homeostatic scaling of synaptic conductances (both glutamatergic and GABAergic) for each astrocytic microdomain was determined independently by the time-averaged activity of glutamatergic synapses in it (according to Equations 11,12).

Several computational models were developed to describe interactions between astrocytes and synapses [35], [36], [37]; however, these models linked increased levels of synaptic activity to calcium elevations in astrocytes, and thus cannot explain how low levels of synaptic activity could culminate in astrocytic release of TNFα. Rather than attempting to develop a detailed mathematical model to describe this process, we assumed here that the ultimate effect of astrocytic microdomain activation is to scale synaptic conductances according to Equations 11,12. This approximation allowed us to avoid introducing additional complexity associated with biochemical cascades of activation in astrocytes [37] and to focus on the long-term network effects of interactions between neurons and astrocytes.

**Figure 4:**
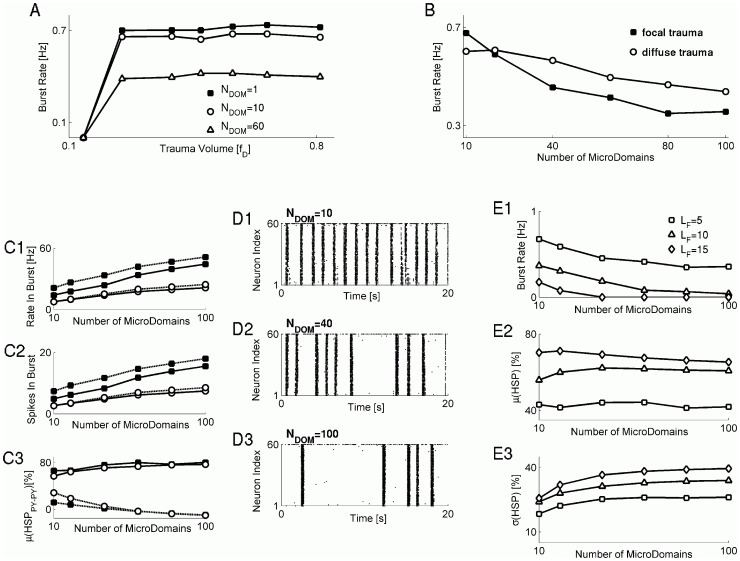
Functional segregation of synaptic input can alleviate the rate of paroxysmal discharge in post-traumatic networks. **A** Burst rate vs the trauma volume, for different number of microdomains for synaptic input segregation: 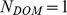 (closed squares, the baseline model); 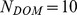 (open circles); 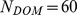 (open triangles). Focal trauma scenario was simulated here. **B** Burst rate vs the number of microdomains, for a fixed trauma volume (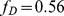): Focal trauma (closed squares); Diffuse trauma (open circles). **C C1**: Mean intra-burst firing rate (average over all bursts and all neurons) vs the number of microdomains: PY neurons (solid lines); IN neurons (dashed lines). Symbol keys and trauma volume are the same as in **B**. **C2**: Mean number of spikes fired during the burst (average over all bursts and all neurons) vs the number of microdomains. All keys and trauma volume are the same as in **C1**. **C3**: Mean of HSP scaling factor at PY-PY synapses, computed separately for synapses arriving from deafferented model PY neurons (solid lines) and from intact model PY neurons (dashed lines), plotted vs the number of microdomains. Closed squares: focal trauma. Open circles: diffuse trauma. Trauma volume parameter was 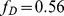. **D** Sample raster plots demonstrating the effect of microdomain parititoning on the rate of paroxysmal discharges: 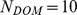 (**D1**); 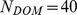 (**D2**); 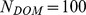 (**D3**). **E** Burst rate (**E1**), mean (**E2**) and standard deviation (**E3**) of HSP scaling factor at PY-PY synapses, vs the number of microdomains, plotted for different sizes of synaptic footprint: 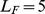 (squares); 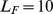 (triangles); 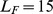 (diamonds). In plots **B–E**, the trauma volume parameter was 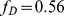.

Figure 4A shows the dependence of paroxysmal discharge rate on the trauma volume, for several scenarios in which synaptic input to each model PY neuron was partitioned into several microdomains. We considered here scenarios of focal trauma. For values of trauma volume above a critical threshold, the burst rate still did not depend on the volume of trauma but the plateau value of paroxysmal burst rate now depended on the number of microdomains into which synaptic input set was partitioned. When plotted vs. the number of microdomains (for the same trauma volume), the burst rate monotonically decreased for larger numbers of microdomains (Figure 4B), and reached an asymptotic level of ∼0.3 Hz for 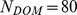 microdomains, corresponding to a situation in which each synapse to a PY neuron was associated with an unique microdomain (each model PY neuron received, on average, 55 synapses from its fellow PY model neurons, and the maximal number of glutamatergic synapses per PY neuron was 75). For values 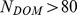, some microdomains had zero “synaptic occupancy” (i.e., they had no synapses to regulate) and thus did not take part in homeostatic synaptic plasticity. Notably, for the same trauma volume, the burst rate that emerged as a result of diffuse trauma also depended on the number of microdomains, but this dependence was much weaker compared to that of the corresponding focal trauma (Figure 4B, compare closed squares and open circles). Thus, even though diffuse trauma resulted in a lower rate of bursts in the model with one microdomain, for strongly segregated set of synaptic input it resulted in more frequent bursting than did the focal trauma.

The averaged intra-burst firing rates of PY and IN model neurons were higher for stronger segregation of synaptic input into microdomains (Figure 4C1), as were the average numbers of spikes fired by model neurons during the burst (Figure 4C2). Because the firing rates of model PY neurons in our model could influence the outgoing synaptic conductances through the presynaptic part of HSP rule, we also computed the mean HSP scaling factor separately for the set of PY-PY synapses arriving from deafferented model PY neurons and the set of PY-PY synapses arriving from intact model PY neurons. The value of the HSP scaling factor is directly proportional to the value of synaptic conductance after scaling, and thus could be taken as a measure of how much the outgoing synaptic conductance of the deafferented vs. intact model PY neurons changes as a function of the number of microdomains and the pattern of trauma. Figure 4C3 shows that the mean HSP scaling factor shows increasing trend as a function of 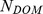 for excitatory input from deafferented neurons, but decreases with larger 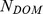 for excitatory input from intact neurons. This effect is qualitatively the same for either diffuse or focal trauma scenarios (Figure 4C3, closed vs. open symbols).

The effect of microdomain partitioning in reducing the rate of paroxysmal bursts was further seen by visual inspection of network activity raster plots (Figures 4D1,2,3). This suggested that the underlying effect of stronger input segregation on paroxysmal burst rate could be similar to that of altered synaptic footprint size (Figure 3A1) – namely, that increased heterogeneity of synaptic input would lead to the decreased burst rate. Indeed, as Figure 4E1 shows, increasing the synaptic footprint size resulted in the downward offset of burst rate, consistent with the results shown in Figure 3A1. Both the mean and the standard deviation of the HSP scaling factor at PY-PY synapses were in general higher for larger synaptic footprint size, for all values of microdomains considered (Figures 4E2,3). The mean value of HSP scaling factor at PY-PY synapses was nearly independent of 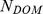, while its standard deviation was generally higher for larger 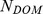 (Figures 4E2,3).

Segregation of synaptic input into microdomains allows a more independent scaling of the inputs from deafferented and intact neurons and thus enhances the correspondence between the firing rate of a given presynaptic neuron and the resulting homeostatic scaling of its downstream synaptic conductance. As a result, in segregated inputs scenario, synaptic conductance from intact neurons is weaker than synaptic conductance from deafferented neurons (Figure 4C3). On the other hand, the postsynaptic component of HSP scales all of synaptic conductances (from both deafferented and intact neurons) by the same amount. Thus, the role of intact neurons in burst generation is weaker (by virtue of their weaker synaptic conductance), but intra-burst firing becomes more intense (partially because of the stronger synaptic scaling in deafferented neurons). Thus, random segregation of synaptic input into microdomains acted to reduce the rate of paroxysmal discharges via a HSP-mediated increase in the variance of the synaptic scaling distribution.

### Morphological remodeling of astrocytes during early post-traumatic period could represent an adaptive response to paroxysmal electrical activity

**Figure 5:**
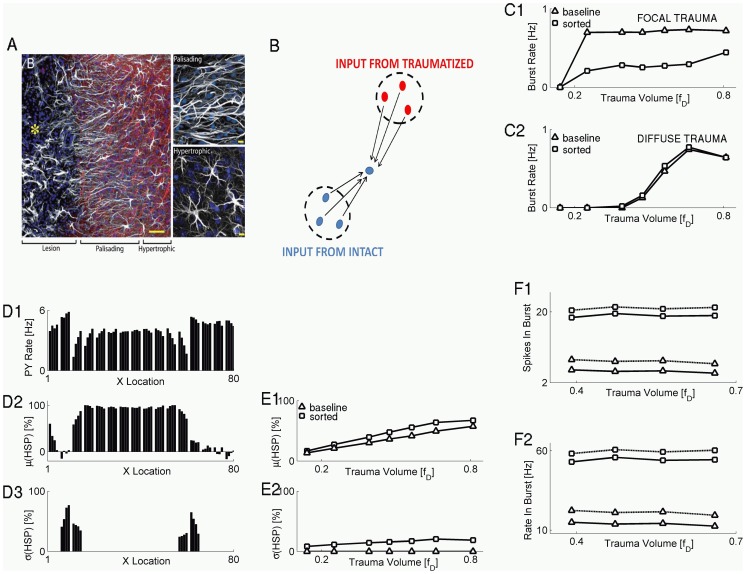
Morphological post-traumatic reorganization of astrocytes can enhance functional segregation and alleviate the rate of paroxysmal activity. **A** Post-traumatic reorganization of astrocytes in a glutamate injury model of cortical trauma. Reproduced with permission from [26]. **B** Schematic presentation of functionally segregated synaptic input. **C** Burst rate vs. trauma volume for focal (**C1**) and diffuse (**C2**) trauma scenarios, for different input segregation scenarios: not segregated input with 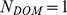 (triangles); segregated input with 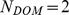 (squares). **D** Distribution of PY firing rates (**D1**), mean (**D2**) and standard deviation (**D3**) of HSP scaling factor at PY-PY synapses. Trauma volume parameter was 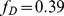. **E** Network-averaged mean (**E1**) and standard deviation (**E2**) of HSP scaling factor at PY-PY synapses vs. trauma volume. **F** **F1**: Number of spikes fired in burst (average over all bursts and all neurons) vs. the trauma volume: PY neurons (solid line); IN neurons (dashed line). Symbol keys are the same as in **C1**. **F2**: Mean firing rate in burst (average over all bursts and all neurons) vs. the trauma volume. All keys are the same as in **F1**.

Results reported in the previous section suggest that functional segregation of homeostatic synaptic scaling by astrocytes has the potential to alleviate the rate of paroxysmal burst discharges in post-traumatic network albeit the reduction in burst rate is relatively small when only a small number of microdomains is considered. Remarkably however, astrocytes in injured cortex also undergo significant rapid morphological remodeling [26]. Specifically, in the ferrous chloride model of trauma-induced epilepsy, astrocytes that were located relatively close (200 microns) to the boundary between intact and injured cortical regions lost their trademark star shape and elongated in the direction perpendicular to the trauma boundary [26]. Accordingly, the astrocytes at the boundary between intact and injured regions were termed “palisading astrocytes”, to distinguish from “hypertrophic astrocytes” that were located in intact part of the network and still retained their “star shaped” morphology to some extent (Figure 5A). Similar reorganization of astrocytes has also been observed in the kainate-induced epilepsy model [26], suggesting that it may represent a generic response of astrocytes to the trauma-induced alterations in neuronal activity. Because astrocytic morphology critically determines its ability to sense synaptic activity (and inactivity) such trauma-induced reorganization might have important implications for post-traumatic epileptogenesis.

To model the role of astrocytic morphological reorganization in trauma induced epileptic like activity, we used the microdomains scheme (as described in Materials and Methods and above) to model the presynaptic component of HSP and further assumed that synaptic input arriving to a specific model PY neuron was subdivided into two microdomains. One microdomain included the synapses exclusively from intact presynaptic neurons, and the other microdomain included synapses exclusively from deafferented presynaptic neurons (Figure 5B for schematic). This was a critical assumption and it derived from the observation that in the experimental model of trauma the “palisading astrocytes” were aligned perpendicular to the boundary separating the intact and traumatized parts of cortical tissue (Figure 5A). We further assumed that this orientation allows for a better segregation of synaptic input into distinct groups (inputs arriving from traumatized neurons vs. inputs arriving from relatively intact neurons). The postsynaptic component of HSP was modeled according to Equations 11,12.

In the focal trauma scenario, the rate of paroxysmal bursts was significantly lower for the scenario of “segregated inputs” (compared to the model with one microdomain), and this difference was observed for a wide range of trauma volumes considered (Figure 5C1). For comparison, in diffuse trauma model, the segregation of synaptic input did not have any significant effect on the burst rate trauma volume relation (Figure 5C2).

Firing rates of PY neurons varied depending on the neuronal location in the network (Figure 5D1, variation of firing rate along X location). The mean (Figure 5D2) and the standard deviation (Figure 5D3) of the PY-PY HSP scaling factor (computed individually for each model PY neuron) also showed strong dependence on neuronal location, with neurons on the intact-traumatized boundary having zero mean HSP and strong variability in synaptic conductances (Figures 5D2,3). The average over the population of model PY neurons of the mean HSP scaling factor was positively correlated with the trauma volume, and its value in the segregated inputs scenario was somewhat higher than that obtained for the model with one microdomain (Figure 5E1). The average (computed over the population of model PY neurons) standard deviation of the HSP scaling factor was also positively correlated with the trauma volume, and its value in the segregated inputs scenario was significantly higher than that obtained for the model with one microdomain (Figure 5E2).

As expected, the reduction in burst rate was paralleled by an increase in the average number of spikes fired per burst (Figure 5F1), as well as by an increase in intra-burst spiking rate (Figure 5F2) of model PY and IN neurons.

## Discussion

Based on the computational model of deafferentation-induced cortical trauma presented here, we predict that the functionally segregated organization of synaptic input to a neuron could help alleviate the rate of paroxysmal activity in early post-traumatic period (hours to days). The post-traumatic morphological reorganization of astrocytes could further mitigate paroxysmal activity [26]. Thus, potential therapeutic approaches to post-traumatic epilepsy should include the influence of astrocytes.

The increased heterogeneity of synaptic organization, reflected in the higher variability of HSP scaling factors at the boundary between intact and traumatized subnetworks (Figure 5E2), may underlie the reduced rate of paroxysmal bursting, which, however, comes at the expense of more intense spiking activity during the burst. In addition, these modeling results suggest that trauma-induced morphological reorganization of astrocytes on the intact-traumatized boundary could further increase the functional segregation of synaptic input to neurons, thus helping to alleviate paroxysmal activity.

### Impact of spatial heterogeneity on paroxysmal activity

In our previous studies we showed that the pattern of trauma can itself determine the threshold for post-traumatic paroxysmal activity [20]. These observations were also qualitatively reproduced in the present study of early post-traumatic reorganization driven by spatially local HSP rule. In particular, in focal trauma scenarios paroxysmal activity emerged for lower values of trauma volume parameter as compared to the diffuse trauma scenarios (Figure 1). In addition, in focal trauma scenario the rate of paroxysmal discharges did not depend on the trauma volume, but showed strong dependence on it in diffuse trauma cases (Figure 1).

A simple explanation of these simulation results would be as follows: In focal trauma and spatially local HSP rule, neurons at the boundary between intact and deafferented regions get ∼50% of their synaptic input from other intact neurons and another ∼50% from deafferented neurons. Because this breakdown does not depend on the trauma volume, and because paroxysmal bursts are generated at the boundary between intact and deafferented regions [20], the burst rate is not expected to depend on the trauma volume. Conversely, for diffuse trauma and local HSP, the breakdown of synaptic input from intact/deafferented neurons monotonically increases (in favor of deafferented neurons) with trauma volume, thus contributing to the increase in burst rate.

We showed earlier [20] that the propagation of paroxysmal events is undermined in diffuse trauma, where asynchronous activity of intact neurons helps to “dissipate” the correlated firing associated with the network burst. Together with the present results, this suggests that the relative role of trauma volume vs. functional segregation of synaptic input depends on the pattern of trauma, with focal trauma allowing for a more efficient “containment” of paroxysmal activity by functional input segregation. We suggest that functional segregation can be achieved by the morphological reorganization of astrocytes, similar to what was observed in some recent experiments [26].

### Novel contribution of astrocytes to post-traumatic epilepsy

It is widely recognized that astrocytes assume a critical role in different kinds of epilepsies [38]. Computational modeling suggested that glutamate signaling from astrocytes to neurons may drive spontaneous neuronal oscillations [35] and give rise to paroxysmal depolarization shifts as often seen in epilepsy [39]. Recent experimental study confirmed these earlier modeling results by directly demonstrating that a positive feedback loop between neurons and astrocytes can drive neurons to seizure threshold [40].

In our own models of post-traumatic epileptogenesis [3], [18], [20], paroxysmal activity is a consequence of homeostatic synaptic plasticity, which is mediated in part by TNFα that is released by astrocytes in response to neuronal inactivity [8]. All of the above mechanisms of astrocytic involvement in epileptogenesis are based on abnormal variations in neurotransmitter/cytokine signaling. By contrast, we showed here that structural reorganization of synaptic input regulation by astrocytes could constitute a contra-convulsive mechanism. It is tempting to speculate that such a seizure-suppressing program is switched on a short time after the traumatic event to compensate for pro-seizure influences of neuroinflammation; however, to fully address this issue, more refined clinical data (showing, for example, the relative timing of TNFα release vs. post-traumatic morphological reorganization of astrocytes) is needed.

The main clinically relevant prediction of our model is that a local homeostatic regulation of synaptic activity by astrocytes can lead to distinct network behavior (compared to the global homeostatic regulatory process). This prediction can be tested by targeting glial fibrillary acidic protein (GFAP), which is a common biological marker of trauma-induced morphological transformation of astrocytes and is dramatically increased during reactive gliosis. GFAP helps astrocytes to maintain mechanical strength and thus is instrumental in determining the cell shape and spatial distribution of finer astrocytic processes (microdomains) for synaptic regulation. Thus, our model would predict a higher incidence of seizures in trauma models of GFAP knockout animals. A higher incidence of seizures should occur following failure of astrocytic microdomains to reorganize after trauma. Consistent with this prediction, one experimental study reported that hippocampi of GFAP knockout mice exhibited higher sensitivity to kainate-induced seizures [41]. We predict that this GFAP deficiency affects seizure susceptibility via failed regulation of synaptic conductance by astrocytic microdomains.

The pathological action of homeostatic synaptic plasticity studied here is only one of the several known mechanisms of epileptogenesis. A common, long-recognized, cause of epileptic seizures is impaired clearance of extra-cellular potassium ions [42], [43], [44]. During intense bouts of neuronal activity extracellular potassium may rise to relatively high levels (10–12 mM) thus further depolarizing the neurons and contributing to the onset of seizure [44], [45]. The predominant mechanism of extracellular potassium clearance is through its reuptake and spatial buffering by astrocytic inward rectifying potassium channels [46]. In fluid percussion model of traumatic brain injury, glial contribution to extra-cellular potassium homeostasis is controversial. Some studies (e.g. [47], [48]) reported alteration in glial uptake of potassium; results of another study suggest that glial contribution to potassium uptake is not altered immediately after trauma [49]. Because the relative apposition of glial potassium channels and neurons is likely to depend on the spatial orientation and cell shape of astrocytes, it is plausible that the spatial pattern of extracellular potassium is further affected as a result of astrocytic reorganization. However, whether reactive astrocytes become more efficient in potassium clearance remain to be shown explicitly.

Trauma-induced morphological reorganization of astrocytes, as well as their release of cytokines and gliotransmitters, is part of “reactive gliosis”, a complex set of processes whereby astrocytes undergo various molecular and morphological changes [50]. Because of its role in the formation of glial scar that prevents axon regeneration, reactive gliosis has been associated with the detrimental effects of trauma-induced reorganization of neural circuitry. However, emerging evidence (reviewed in [50]) indicates that reactive astrocytes can have both beneficial and detrimental effects on post-traumatic reorganization.

### Anti-epileptic drugs and homeostatic plasticity

Management of post-traumatic epilepsy and related convulsive disorders is often done with a variety of anti-epileptic drugs [51]. In particular, phenytoin and/or sodium valproate therapy can prevent early posttraumatic seizures [52], [53]. It was shown that sodium valproate inhibits production of TNFα through inhibition of NF-κB [54]. Extensive experimental data implicating the role of TNFα in seizure generation (reviewed in [16]) and results of our modeling studies thus provide an explanation with regard to the mechanistic action of valproate in early post-traumatic seizure suppression. Interestingly, valproate was found to be less efficient in suppression of late post-traumatic seizures [52]. This may imply that the trauma-induced astrocyte-mediated TNFα signaling possibly represents one out of several pathways of post-traumatic homeostatic regulation that tends to reorganize the network in response to chronic changes in electrical activity. Indeed, other mechanisms, such as trauma-induced changes in anatomical connectivity, have been implicated in post-traumatic epileptogenesis [2], [17], [19], [30], [55]. These mechanisms are likely to operate on much slower time scale than regulation by glial TNFα. The multiple temporal scales of post-traumatic HSP could thus explain the fact that anti-epileptic drugs are more efficient during early post-traumatic period (when they target specific pathway) as opposed to their relative inefficiency during late post-traumatic period (when other HSP pathways are activated).

### Complex involvement of astrocytes in post-traumatic reorganization

Synaptic activity can be increased or decreased by molecules released from astrocytes and vice versa. In particular, accumulating evidence indicates that endocannabinoids (eCB) might be involved in compensatory mechanisms to offset the effects of trauma [56]. Endocannabinoids are released from neurons following intense neuronal activity. Recently it was shown that endocannabinoids released from hippocampal neurons can cause phospholipase C dependent calcium elevation in adjacent astrocytes through activation of astrocytic CB1 receptors [57]. The endocannabinoid-mediated activation of astrocytes stimulated the release of glutamate from astrocytes that further promoted the excitability of postsynaptic neurons. On the other hand, application of endogenous cannabinoid was shown to suppress TNFα formation through inhibition of nuclear factor kappa beta (NF-kB) after traumatic brain injury in a CB1-dependent manner [58]; signaling through this pathway is likely to decrease neuronal excitability. Thus, astrocyte-endocannabinoid interaction can contribute to post-traumatic reorganization in a complex manner. How this interaction contributes to the regulation of paroxysmal activity remains to be investigated [59].

### Conclusions

Consistent with this emerging evidence, and based on our modeling results, we propose two opposing roles for reactive astrocytes in early post-traumatic epileptogenesis. Specifically, we propose that the release of TNFα promotes the generation of paroxysmal bursts, and that a post-traumatic morphological reorganization of astrocytes acts to suppress bursting activity at the expense of less frequent, but more intense, paroxysmal bursts. In this conceptual model the reorganization of astrocytes might be an adaptive step aimed at reducing the pro-convulsive effects of TNFα. Interestingly, in the ferrous chloride model of epilepsy, seizure suppressing drugs led to a significant reduction in the extent of seizure-related morphological transformation of astrocytes [26]. Thus, seizure itself appears to be a causative factor behind astrocytic reorganization. It is conceivable then that different components of reactive gliosis are coordinated in a way that aims to maintain minimal risk of seizures incurred by the activation of astrocytes. Biochemical dissection of these components and relations between them may help in understanding the causes of post-traumatic epileptogenesis.

## Materials and Methods

The core computational model used in the present study was based on the modeling framework that we earlier developed to study the effect of different trauma patterns on the incidence of network bursts in post-traumatic cortex [20], [28]. Since in the present work we were specifically interested in the transformation of electrical activity during the early phase of post-traumatic response (when synaptic scaling might be constrained by a local glial response), the computational model of homeostatic synaptic plasticity in post-traumatic cortex needed to be adjusted to reflect the spatial localization of HSP. These changes, and the rationale behind them, are described in details below.

### Computational model of cortical network

**Figure 6:**
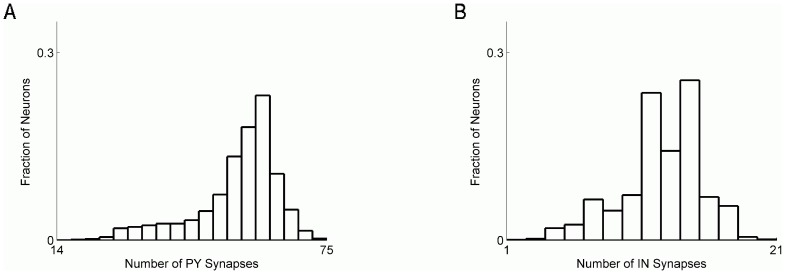
Distribution of synapse number per model pyramidal neuron. **A** Distribution of excitatory (PY) synapses. **B** Distribution of inhibitory (IN) synapses.

A cortical network was modeled as a 2D network on square lattice (80×80 neurons) in which each neuron could establish synapses with its peers with probability 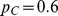 within its local footprint (a square 10×10 neuron region from and to which a neuron could receive or send synaptic connections). Model pyramidal neurons received, on average, 55 synapses from other model PY neurons and 12 synapses from model IN neurons. The distribution of synaptic inputs to model PY neurons is shown in Figure 6. The synaptic footprint of each neuron was centered on the neuron, and the size of the footprint was given by dimensions of the corresponding square region. Pyramidal neurons accounted for 80% of network population (5120 out of 6400 neurons), and inhibitory neurons constituted the remaining 20% (1280 out of 6400 neurons). Inhibitory neurons were distributed regularly on the lattice, such that every 5^th^ neuron was inhibitory. Our goal was to understand the general responses of a cortical network to traumatic perturbation and therefore specific features pertaining to specific cortical layers were not included in the model.

The dynamics of neurons were modeled with the one compartmental Morris-Lecar model [60], as described in detail elsewhere [20], [28]. Briefly, the equations that governed the neuronal dynamics were:
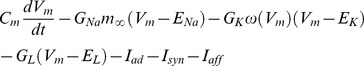
(1)

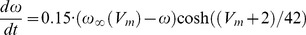
(2)

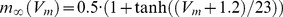
(3)

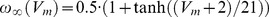
(4)Phenomenological spike frequency adaptation current was added to model PY neurons to account for the experimentally observed spike frequency adaptation:
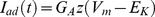
(5)

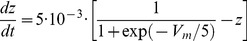
(6)Synaptic currents at PY-PY synapses had both AMPA and NMDA components, with both AMPA and NMDA conductance attenuated by synaptic depression as described below. Inhibitory synaptic currents did not incorporate synaptic depression.

Synaptic transmission was modeled as a deterministic process, during which synaptic conductance rose instantaneously following the spike and relaxed, with the characteristics time 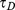, to zero:
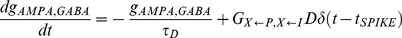
(7)The value of maximal synaptic conductance,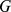, depended on the pre- and postsynaptic neurons. Thus, 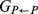 (maximal synaptic conductance from pyramidal neuron to pyramidal neuron) was different from 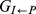 (maximal synaptic conductance from pyramidal neuron to inhibitory interneuron). Values of synaptic conductance are given in Table 1.

**Table 1:** Biophysical parameters.

Peak sodium channel conductance	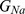	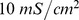
Peak potassium channel conductance	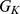	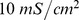
Peak leak conductance	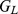	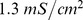
Peak adaptation conductance (pyramidal neurons only)	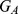	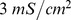
Sodium reversal potential	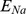	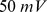
Potassium reversal potential	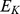	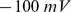
Leak reversal potential	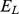	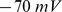
Membrane capacitance	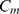	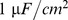
Fast NMDA current decay time	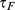	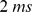
Slow NMDA current decay time	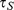	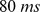
AMPA current decay time	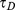	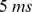
GABA current decay time	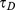	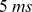
Recovery time from short-term synaptic depression	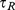	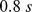
Strength of synaptic depression	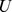	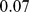
AMPA, NMDA reversal potentials	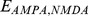	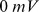
GABA reversal potential	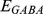	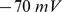
P-to-P peak synaptic conductance (initial value)	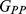	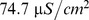
I-to-P peak synaptic conductance (initial value)	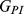	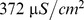
P-to-I peak synaptic conductance	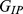	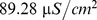
I-to-I peak synaptic conductance	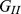	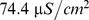
Peak afferent synaptic conductance	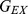	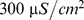
Afferent current decay time	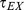	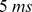
Afferent current reversal potential	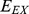	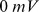
Afferent stimulation rate (intact conditions)	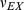	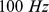
Rate of convergence, homeostatic adjustment	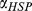	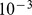
Target firing rate of PY neurons	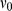	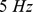

The temporal dynamics of the NMDA conductance was modeled as a difference of fast (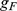) and slow (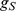) exponentially decaying components:
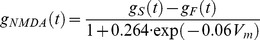
(8)

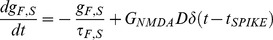
(9)The parameter 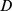 accounted for the efficacy of synaptic transmission. For GABAergic synapses, this parameter was held fixed (D = 1). For excitatory AMPA and NMDA synapses, it evolved according to the following equation, with 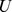 representing the strength of synaptic short-term depression:
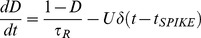
(10)Values of parameters are given in Table 1. In the intact network with no deafferentation model pyramidal (PY) and inhibitory (IN) neurons fired with average rates of 5 and 10 Hz, respectively.

### The deafferentation model of cortical trauma

In addition to network current, each model neuron received an excitatory current 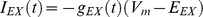 from “the rest” of the cortex (afferent excitation). Synaptic conductance of this current evolved according to: 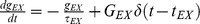, with 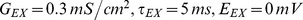. This synaptic conductance was stimulated randomly at times 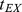 at the baseline Poisson rate of 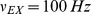.

Trauma was modeled as cortical deafferentation, following which the frequency of external (afferent) excitation to a fraction 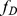 of model neurons (both PY and IN) was reduced to 10% of its value in the intact model (from 100 Hz in intact model to 10 Hz in the deafferented state). The parameter 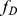 represents the fraction of deafferented (injured) neurons and can be thought of as being proportional to the relative volume of deafferented neurons; thus, we refer to this as the “volume of trauma” parameter. As in our previous studies, we considered scenarios of focal and diffuse trauma. In focal trauma, the deafferented neurons were organized in a contiguous block (Figure 1A1, right). In diffuse trauma, the deafferented neurons were picked up at random from the entire model network (Figure 1A2, right).

### Modeling of post-traumatic homeostatic synaptic plasticity

Recent experimental evidence indicates that TNFα is a permissive, rather than instructive, mechanism that allows the synapses to express homeostatic synaptic plasticity [61]. Thus, our present implementation of HSP aimed to account for both the possible differential nature of up- vs. down- regulation of synaptic conductance and for the permissive (rather than instructive) nature of TNFα signaling. Specifically, we assumed the following mathematical form for the HSP at AMPA synapses of PY-PY pairs:
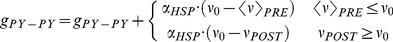
(11) In Equation 11, 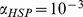 is the convergence rate of the homeostatic update and 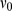 is a preset “target rate” of pyramidal neuron firing (our HSP equation was only applied to model pyramidal neurons), typically set at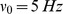. Upregulation of excitatory synaptic conductance on model PY neuron could occur if the average firing rate 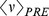 in the set of presynaptic partners (collateral pyramidal neurons that project synapses to a given neuron) was below the preset target rate. This regime corresponds to the release of TNFα from glial cells, which is believed to be determined by the levels of synaptic activity in the proximity of these cells (our implicit assumption is that synaptic activity scales with the firing rate of neurons that generate this synaptic activity). By contrast, downregulation of excitatory synaptic conductance on model PY neuron could occur if the firing rate 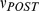 of a neuron under consideration was above the preset target rate.

**Figure 7:**
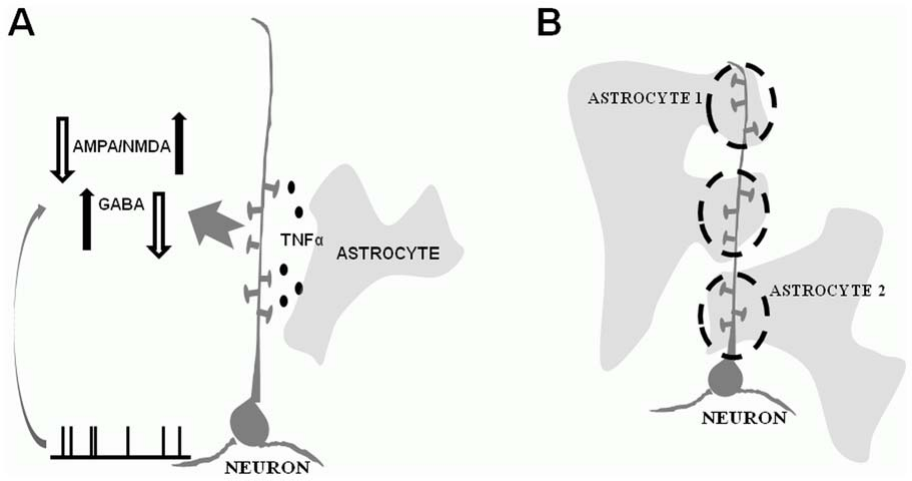
Computational model of homeostatic synaptic plasticity and microdomains. **A** Schematic presentation of homeostatic synaptic plasticity rule used in the baseline version of the model. Upregulation of synaptic conductances to a neuron was determined by averaging firing rates over the set of synaptic inputs to the neuron under consideration. Downregulation of synaptic conductances to a neuron was determined by the firing rate of this neuron. **B** Schematic presentation of input segregation into microdomains, where upregulation of synaptic conductances belonging to the same microdomain was determined by averaging over firing rates of neurons that projected these synapses. Downregulating mechanism of synaptic conductance was the same for all synapses (as it was determined by the firing rate of their common postsynaptic neuron).

This modeling assumption stems from the fact that TNFα can only upregulate excitatory synaptic conductance; thus, down regulation of synaptic conductance likely depends on the firing rate of postsynaptic neuron [5]. It is also consistent with earlier findings regarding the role of neuronal activity in downregulation of synaptic conductances onto it [62]. The update scheme for GABA synapses on model pyramidal neurons is the opposite of that of the AMPA synapses on model pyramidal neurons (the average over the presynaptic set was still taken over the presynaptic set of PY model neurons)
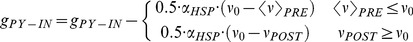
(12)Schematic presentation of homeostatic plasticity rules for up- and down regulation of synaptic conductances is given in Figure 7A.

The extent of per synapse homeostatic adjustment was computed as percentage of change in synaptic conductance relative to its value in the corresponding intact model. Taking PY-PY synapse as an example, 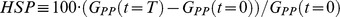 where T is the time after the network had reached its new post-traumatic steady state.

### Modeling functional segregation of synaptic input into microdomains

In some simulations, we investigated the effect that functional segregation of synaptic input into microdomains might have on the post-traumatic reorganization of electrical activity in cortical network. For each model PY neuron, the entire set of 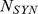 synapses to it was randomly and equally partitioned into 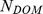 groups, each group on average consisting of 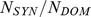 synapses. Cortical neurons receive ∼8,000 synapses [63]. In the model network, each neuron received on average ∼80 synapses. Thus, each model synapse can be thought of as representing a group of ∼100 synchronously activated biological synapses, consistent with small (∼1%) amount of synchrony present at any time in cortical input [64]. The number of microdomains, 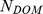, was varied from 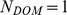 (corresponding to the case when all synaptic input is lumped into single astrocytic microdomain) to 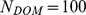 (corresponding to the case when each microdomain is associated with at most 1 model synapse). It was shown that in the cortex, each astrocytic domain can cover up to 80 microns of dendritic length [65]; taking linear spine density 1.0–1.5 spines/micron [66], it follows that each domain can be associated with up to ∼120 synapses. Thus, the case 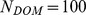 (1 model synapse per microdomain, 1 model synapse = ∼100 biological synapses) yields values consistent with the experimental findings (up to ∼120 biological synapses per microdomain). A schematic diagram for the case 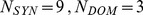 is shown in Figure 7B. The presynaptic component of HSP was computed for each one of these microdomain groups separately, as in Equations 11,12, with 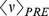 being the firing rate averaged over all model PY neurons that belonged to that same microdomain. Downregulating component of HSP was computed as in Equations 11,12, based on the firing rate of postsynaptic neuron.

Several models of astrocyte-synapse interaction [35], [36], [37] have linked increased levels of synaptic activity to astrocyte calcium elevation. Here, we focused on astrocytic responses to synaptic inactivity. Thus, existing models cannot explain post-traumatic activation of astrocytes. Rather than attempting to develop a detailed mathematical model to describe the response of astrocytes to synaptic inactivity, we assumed here that the ultimate effect of astrocytic microdomain activation was to scale synaptic conductances according to Equations 11,12. This assumption allowed us to avoid introducing additional complexity associated with biochemical cascades of astrocyte activation [37] and to focus on the long-term network effects of neuron-astrocyte interaction.

In a separate set of simulations we modeled correlated segregated inputs, whereby the entire set of synapses to each model PY neuron was partitioned into two groups (two microdomains, 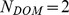) with synapses in one microdomain associated with the inputs from deafferented neurons, and synapses in another microdomain associated with the inputs from intact neurons.

### Detection of paroxysmal bursting events

Following deafferentation, homeostatic synaptic plasticity transformed asynchronous spiking to paroxysmal bursts. Thus, we adopted the rate of paroxysmal burst generation as a measure of network's propensity to exhibit the transition to seizures. Paroxysmal bursts of highly correlated population activity in our model resembled interictal epileptiform discharges (IEDs), which are often considered as an important diagnostic feature of epileptic seizures [22], [23], [24]. Thus, a higher rate of population bursting in a post-traumatic network model was considered as an indicator of stronger propensity to seizures.

Paroxysmal bursting events were detected as previously described in [20]. First, the entire simulation time period was partitioned into non-overlapping bins of 100 milliseconds each. Based on this binning, the spike count per bin for each model neuron was obtained. A paroxysmal bursting event was registered in time bin 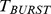 if at least 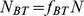 of recorded model neurons (both pyramidal neurons and interneurons) fired action potentials during this time bin, with an average rate of firing greater than the threshold rate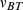. This operational definition was constrained by the minimal fraction of active neurons, 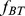, that defined the paroxysmal burst, and by the minimal intra-burst firing rate, 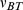, of these active neurons. We set 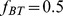 and 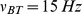.

The large size of our model network (6,400 neurons) made analysis of the entire network computationally intractable; thus, we sampled the activity of a subset of neurons (both pyramidal neurons and interneurons, from both deafferented and intact regions), using this sample to estimate the rate of paroxysmal bursts in the network. In the case of a diffusely traumatized network (spatially random deafferentation), the sampling region was a square block (usually 20×20 model neurons) co-centered with the center of the 2D network because spatially random deafferentation resulted in activity roughly symmetric with respect to the center of the lattice. In the case of a focally traumatized network (spatially structured deafferentation), the sampling region was composed of 5 parallel adjacent lines (resulting in a total of 400 model neurons) because the activity generally propagated from intact regions into deafferented regions.

### The boundary between deafferented and intact parts of the network

In our earlier studies we showed that, in the deafferentation model of post-traumatic epileptic activity, the paroxysmal activity is generated by model pyramidal neurons with partially intact connectivity that are located at the boundary between intact and deafferented regions [20]. Consequently, activity generated at the boundary by intact neurons takes the shape of paroxysmal bursts (waves) that propagate into the deafferented part of a network. In the present study, we quantified some characteristics of network organization (e.g., mean and standard deviation of HSP scaling factors) and neural activity (e.g., cross-covariance between activities of different neurons) at the network boundary. The boundary was operationally defined as a set of intact neurons located within one synaptic footprint from the boundary deafferented neuron.
